# Cancer control is not a one-time event: why screening must become a life-course system

**DOI:** 10.3389/fmed.2026.1800855

**Published:** 2026-03-17

**Authors:** Waseem Jerjes, See Chai Carol Chan, Azeem Majeed

**Affiliations:** Department of Primary Care and Public Health, Faculty of Medicine, Imperial College London, London, United Kingdom

**Keywords:** cancer control, cancer screening, early detection, health inequalities, life-course approach, preventive care, primary care, screening participation

## Introduction

In 2022/23, more than 341,000 people were diagnosed with cancer in England (548 per 100,000) (1). This burden makes prevention and earlier detection essential, yet progress remains uneven. Here, early detection is used as an umbrella term covering both population screening (testing asymptomatic people in defined groups) and early diagnosis (timely recognition and investigation of symptomatic disease). Outcomes have improved for some cancers, but several—including pancreatic and liver cancers—still have poor survival and are commonly diagnosed late, reflecting the absence of population-based screening programmes and the challenges of early detection. Lung cancer also continues to have poor survival and late presentation, despite the introduction of targeted screening for people at high risk. Accordingly, this article focuses on improving the delivery and completion of established screening pathways (from invitation to action and follow-up), rather than reviewing symptom-based early diagnosis strategies for cancers without population screening. The gap is increasingly one of delivery: how prevention, screening and follow-up are designed and implemented at scale.

Routes-to-diagnosis data suggest that around 55% of cancers are diagnosed outside screening programmes or urgent suspected cancer (USC) referrals (the “2–week-wait” pathway from primary care) (2). Over the past 5 years, USC referrals have converted to cancer at around 6–7% nationally (3). These figures are interpreted differently. Some interpret low conversion as over-referral; others argue it reflects appropriately low thresholds when the cost of missed cancer is high (3). Either way, the current model generates high urgent activity while many cancers still emerge through other—often later—routes. When detection depends on incidental encounters, it is clinically inefficient and can feel arbitrary to patients.

The UK's national screening programmes deliver measurable benefit and remain among the most important population health interventions the NHS provides. NHS England estimates that cervical screening saves around 4,500 lives each year; breast screening prevents around 1,300 deaths and detects around 19,000 cancers annually; and regular bowel screening reduces the risk of dying from bowel cancer by at least 25% (4). These are major achievements. Screening also carries predictable trade-offs—false positives, overdiagnosis, anxiety, and downstream capacity demands. A life-course model should therefore be designed to increase benefit while minimizing harm, including clear communication of risk, proportionate recall, and rapid resolution of abnormal results.

In England, the main national screening programmes (breast, cervical and bowel) are organized through registry-based call-and-recall systems that invite eligible people directly, with follow-up distributed across services. General practice does not “run” these programmes, but it is where patients seek advice, where competing priorities are managed, and where non-completion and abnormal results can be actively followed up. This separation between programme delivery and continuity-based care helps explain why invitations can be issued at scale while completion remains uneven.

Yet participation remains below the level usually required for maximal population impact. Breast screening participation is around 70%, bowel screening around 72%, cervical screening around 68–75%, and lung screening can be as low as 20% in some settings (5–7). These shortfalls are not unique to England. Internationally, many high-income countries report screening participation that falls short of optimal levels, and the pattern of unequal uptake across social groups is persistent (8, 9). In England, uptake is also patterned by deprivation and local context, and UK qualitative evidence highlights practical barriers (time, access), emotional barriers (fear, embarrassment), and low perceived need—supporting the case for active follow-through rather than invitation-only models (10, 11). A system that depends on high participation but consistently delivers unequal participation will struggle to maximize population benefit.

We argue for a shift from episodic, age-triggered screening to a life-course approach: a closed-loop delivery system that recognizes cumulative risk, uses timed invitations and recall aligned to life circumstances, and measures completion from invitation to definitive action. This is not a call to abandon age-based programmes, but to strengthen them by embedding equity-focused follow-through and risk-aware timing.

The rationale is pragmatic. Low screening impact is not only about people choosing not to participate; it is also about mis-timing, meaning that programmes deliver invitations when individuals are less able or less ready to respond. A system can “offer” screening and still fail to deliver it if it is not timed to real life and does not support follow-through. In primary care, non-completion often reflects predictable life pressures and service friction—for example pregnancy/postpartum disruption to cervical attendance, confusion or stigma around lung screening, and bowel kits that arrive without active recall or practical support. These examples are illustrative, but consistent with evidence that isolated “send-and-forget” offers tend to plateau and amplify inequalities. The question is therefore not whether screening “works” in principle, but how to design a system that works in practice across decades, across life circumstances, and across social gradients.

## Rethinking screening as a life-course system rather than an age-based event

Cancer risk is not created at the moment a screening letter arrives. It accumulates over time through exposures and contexts that begin long before most age-based thresholds. Risk accumulates through exposures and contexts over decades, including smoking, alcohol, obesity, metabolic risk, and the social determinants that shape them (8, 9). The challenge for screening programmes is that age captures only a slice of this reality. Two people of the same age can have profoundly different risk trajectories and profoundly different capacities to engage with preventive healthcare. If programmes remain structured around age alone, they will systematically miss opportunities to intervene earlier for higher-risk groups, and they will repeatedly deliver invitations when people are least able to respond. These are not marginal issues; they are central to why participation plateaus below the desired thresholds and why inequalities persist despite repeated campaigns.

The International Agency for Research on Cancer has emphasized that cancer prevention must be approached as a long-term public health endeavor grounded in risk reduction and systems, not solely as a technological challenge (10–12). It has also proposed essential and desirable criteria for organized cancer screening programmes, highlighting the importance of population identification, quality assurance, equity, informed choice, and timely referral and follow-up (13). A lifetime approach translates that prevention logic into delivery. It does so by treating screening as part of a continuous programme that runs alongside people's lives, rather than as a periodic transaction that depends on individuals repeatedly overcoming barriers without support.

One way to make this concept concrete is to compare it to other chronic-risk models that health systems already accept. Lifelong hepatitis B control offers a clear example. Universal infant vaccination, treatment of chronic infection, and six-monthly surveillance of high-risk adults have reduced hepatitis B surface antigen prevalence, lowered hepatocellular carcinoma incidence, and shifted detection toward more treatable stages (14). This model is not defined by age alone. It is defined by risk and timing, with surveillance intervals and triggers anchored to infection status and risk. It also assumes follow-up as a core feature, not as an optional add-on. The success of this approach illustrates what becomes possible when a health system accepts that prevention is not a one-off event.

The WHO strategy to accelerate the elimination of cervical cancer similarly reflects life-course thinking: HPV vaccination in adolescence, adult screening to detect precancer, and treatment to prevent progression (15). Although age bands exist within these strategies, the underlying logic is sequential and sustained across life stages. The key point is that elimination thinking treats prevention as a programme that follows cohorts through time. It is inherently systems-oriented.

A lifetime approach to broader cancer control does not imply blanket surveillance. It implies better use of what health systems already know and what primary care already does. It involves continuous risk capture, timely invitations linked to risk and life stage, and a closed-loop process that measures completion rather than distribution of letters or kits. In practical terms, it means that an invitation is not the endpoint of programme delivery; it is the start of a measurable pathway. Table 1 summarizes a practical “lifetime cancer control” blueprint, illustrating how life-stage triggers, patient experience, primary care action, service infrastructure and policy accountability can be aligned into a closed-loop system.

**Table 1:** A practical “lifetime cancer control” blueprint for implementation across patients, clinicians, services and policy.

**Life-stage window or trigger (not age-only)**	**What the system detects (risk/context)**	**What the patient experiences (offer and support)**	**What the clinical team does (pathway ownership)**	**What services/IT must enable (closed-loop design)**	**What policy should measure (accountability)**
Pregnancy and postpartum period	Overdue cervical screening; competing priorities	Planned deferral with timed re-invite; flexible appointments; supportive messaging	Endorsement when appropriate; structured recall task rather than “missed opportunity”	Ability to pause, schedule, and re-invite; supported booking	Completion within defined postpartum window; equity by deprivation/ethnicity
Smoking status change (current smoker or recent quitter)	Lung risk inflection; stigma risk	Non-judgemental framing; navigation support; clear explanation	Brief clinician explanation; address confusion; agreed plan to completion	Tailored communications; outreach calls; mobile/out-of-hours options	Completion and patient understanding; stage shift in high-risk cohort
Non-completion after bowel screening invitation	Missed step recorded	Practical support and reminders; reduced friction to complete	Team-led follow-up; clinician endorsement during any routine contact	Registry-linked reminders and task lists; escalation rules	“Recovered completion” after non-response; interval cancer monitoring
New first-degree family history coded	Increased hereditary risk	Personalized risk conversation and earlier pathway	Risk counseling; referral/genetic assessment where indicated; earlier recall	Templates and referral pathways integrated with record	Timeliness of risk assessment; completion of surveillance pathway
Rising metabolic risk (BMI/HbA1c)	Cumulative risk trajectory	Prevention discussion linked to timely screening completion	Integrate screening tasks into chronic disease reviews	Automated prompts; co-ordinated booking and tracking	Uptake and completion in higher metabolic-risk groups; inequality gap
Menopause/HRT reviews	Life-stage transition; preventive opportunity	Co-ordinated screening plan with clear next steps	Align screening and follow-up; address concerns	Integrated scheduling and results tracking	Completion and time-to-action; patient experience
Becoming a carer or undergoing social-care assessment	Access barriers; high competing demands	Flexible options including out-of-hours/mobile; supported booking	Identify barriers early; route to navigation	Outreach capacity; flexible commissioning models	Uptake among carers; reduction in late-stage presentation
Moving GP practice/new registration	Risk of being “lost” to follow-up	Catch-up plan at registration	Confirm overdue actions; set recall tasks	Data transfer and registry reconciliation	Reduced missed invitations at transition points

## Primary care as the engine for lifetime cancer control

Primary care sits across the life course. It sees people during adolescence, before and after pregnancy, through mid-life risk accumulation, and into later-life multimorbidity. It holds longitudinal information about behaviors, comorbidities, family history, medicines, and social context (15–17). If cancer control is to become genuinely life-course, primary care is the most plausible platform to operationalise it, not because it is the only place prevention happens, but because it is the place where continuity can transform a generic offer into a supported pathway.

A practical lifetime model would embed structured prevention and screening prompts within routine primary care touchpoints, particularly those that already exist and already have policy recognition (15). NHS Health Checks, for example, are a structured mid-life risk assessment programme designed to identify modifiable risk factors and support preventive interventions systematically (16). As a policy-recognized touchpoint, they also provide a practical opportunity to link risk-factor management with supported screening completion when appropriate. Yet in many settings, they remain siloed from cancer prevention and screening completion. Similarly, routine consultations for chronic disease reviews, repeat prescription renewals, contraception care, menopause or HRT reviews, and postnatal checks are predictable moments when people are already engaging with healthcare. These moments represent opportunities not for opportunistic “by the way” screening reminders, which add cognitive burden to clinicians, but for protocolised, team-based pathways that prompt, support and follow through.

The Royal College of General Practitioners has highlighted the central role of primary care in cancer diagnosis and provides practical tools to improve recognition, safety-netting, and referral decisions (17). A lifetime model should be seen as complementary to this agenda. Screening will never detect all cancers. Symptomatic routes will remain essential. A life-course approach strengthens prevention and follow-up so that fewer diagnoses depend on opportunistic encounters or delayed escalation. In this model, the same system that ensures screening completion also strengthens safety-netting for symptoms, because it builds the habit of tracking an issue to closure rather than allowing it to dissipate into “missed steps”.

A common objection is capacity. General practice is already under strain. The answer cannot be to add a long list of additional clinician tasks. The answer must be to redesign delivery so that the work is distributed and supported. A lifetime model needs clear division of labor. Clinicians provide endorsement, judgement for complexity, and clinical decision-making where there is uncertainty. Administrative staff, care coordinators, social prescribers, health coaches, and navigators provide practical follow-through, supported by interoperable systems that generate tasks and reminders. In other words, the model should be built to reduce clinician workload per completed screening journey, not increase it.

## The patient experience: why timing and completion matter as much as invitation

When screening participation is discussed, the conversation often drifts toward the language of “uptake” as though it were a simple behavioral choice. For patients, however, screening is not a statistic; it is a lived process. They fail to participate because the invitation arrives at the wrong moment, because the next steps are not clear, because fear or stigma interferes, because practical obstacles are not addressed, or because previous negative experiences make avoidance rational.

Cervical screening illustrates timing and competing priorities. The system can technically “invite” someone, yet deliver the invitation during pregnancy or early postpartum when the individual is juggling multiple health appointments, childcare, fatigue, and sometimes recovery from traumatic birth experiences. A lifetime approach does not interpret non-response as an endpoint. It treats non-response as expected and builds planned recall to a more feasible window, while offering flexible appointments and appropriate communication. The shift is from a system that sends and forgets to a system that pauses and returns at the right time.

Lung screening illustrates stigma and meaning. Screening offers can be experienced as blame, particularly where people already carry shame about smoking or where health literacy barriers make the purpose of screening unclear. Low uptake in lung screening, sometimes reported around 20%, (5) is therefore not surprising. A lifetime model would not rely on the screening programme alone to communicate. It would incorporate clinician-supported explanation and reassurance, and it would offer navigation support to address practical barriers. The invitation would be framed as a supportive health offer linked to risk and benefit, rather than as a judgement of personal choices.

Bowel screening illustrates the gap between provision and completion. A kit posted through the letterbox may be easy to distribute but not easy to complete. People may feel uncertain, embarrassed, or simply overwhelmed by instructions. Without clinician endorsement and without active recall, a substantial proportion will ignore or postpone the kit. A lifetime model would incorporate brief endorsement at routine contacts, task-based follow-up by the practice team, and structured re-invitation if the pathway is incomplete. This does not remove personal choice; it makes completion achievable.

International comparisons reinforce that these issues are structural rather than cultural quirks. The OECD and Eurostat data show that screening participation varies widely and often falls below optimal targets, even in well-resourced systems (8, 9). The common thread is that programmes struggle when they are designed as isolated events rather than as supported pathways integrated with trusted care relationships and registry-linked follow-up.

## Service and policy implications: from “coverage” to accountable pathways

A lifetime approach is not simply a clinical philosophy; it is a service and policy proposition. It requires infrastructure that many health systems partially possess but do not consistently use for cancer prevention.

The first requirement is registry linkage and interoperability. Because this approach uses personal data more actively, it must be underpinned by transparent governance, clear accountability for data quality, and public trust—particularly for risk-stratified invitations. A closed-loop model is impossible if primary care cannot reliably see invitation status, attendance, results and follow-up requirements across services. Programmes need to move beyond counting invitations to tracking completion and time-to-action. This is partly technical and partly governance. It requires agreement on who owns which step of the pathway and how missed steps are escalated.

The second requirement is explicit trigger design that goes beyond age. Where risk-stratified invitations are considered, they should be cancer-specific and introduced only where evidence supports net benefit, acceptable harms, and equitable delivery (13). The triggers should be simple enough to be operational, auditable, and equitable. Examples include a newly coded first-degree family history of cancer; a change in smoking status; the development of metabolic risk such as rising BMI or HbA1c; initiation of long-term medicines associated with increased risk; entry into pregnancy and the postnatal period; or major transitions such as moving practice or becoming a carer. The point is not to generate endless prompts, but to place screening and prevention at moments when risk is changing or readiness is higher. In practice, triggers should be few, prioritized, and auditable, with safeguards to avoid alert fatigue and unintended widening of inequalities.

The third requirement is navigation capacity. If the system continues to rely on patients to self-navigate appointments, transport and follow-up, it will continue to deliver unequal outcomes. Navigation is not an optional “nice to have”. It is the equity mechanism that translates an invitation into a completed preventive action, particularly for people with unstable work, caring responsibilities, language barriers, disability, or lower health literacy. Navigation can be delivered through primary care teams, community outreach, and flexible service models such as mobile units and out-of-hours clinics. Without commissioning flexibility, navigation cannot operate at scale.

International examples show that integrated, registry-driven models can sustain participation. Australia's National Cervical Screening Program has expanded self-collection, supported by policy and registry infrastructure, which can help people who are reluctant or unable to attend traditional screening (18). The Netherlands runs a registry-linked system that sequences invitations and reminders across programmes and supports GP follow-up and self-sampling options, with participation commonly above 70% (19). These systems are not perfect and do not always reach the ideal 80% threshold, but they illustrate what can be achieved when screening is treated as an organized pathway rather than a single transaction.

Policy should therefore align incentives and accountability with completion and equity, not just with activity. In practical terms, commissioners and national bodies should ask: what proportion of invited individuals complete screening? How many complete after initial non-response when navigation support is offered? How long does it take for abnormal results to reach definitive action? How does completion differ by deprivation, ethnicity, language, disability and mental health status? A high-performing system measures these questions routinely and adapts delivery accordingly.

## Discussion

Cancer screening has saved lives and remains essential, but the way programmes are delivered still leaves predictable gaps in completion and equity. England's screening programmes demonstrate clear benefit, (4) but participation remains below optimal levels in breast, bowel, cervical and lung screening (5–7). Meanwhile, a substantial proportion of cancers continue to be diagnosed outside screening and USC pathways (2). The conversion rate of USC referrals, around 6–7%, (3) highlights the tension between sensitivity and specificity in urgent investigation, but it also underscores a broader point: a system that leans heavily on urgent referrals and incidental findings will remain reactive rather than preventive.

A lifetime approach offers a coherent, testable alternative. It treats cancer control as a long-term programme aligned to cumulative risk and life-stage windows of receptivity, delivered through continuity-based care, and measured through completion and stage shift in higher-risk subgroups. It is grounded in the logic of prevention advanced by international cancer prevention frameworks, (12) and supported by analogies to successful long-term risk control programmes such as hepatitis B vaccination, treatment and surveillance (14). It also aligns with global life-course strategies such as cervical cancer elimination through HPV vaccination and sustained screening (15).

Primary care provides the natural infrastructure for this model, because it can combine continuity with routine risk capture and planned recall (15, 16). The model does not ask clinicians to do more unsupported work; it asks the system to redesign delivery so that prevention and screening become closed-loop pathways supported by registries, navigation capacity and flexible service models. Tools and frameworks already exist in primary care for structured prevention and improved cancer diagnosis, (16, 17). but they need to be integrated into a coherent life-course approach rather than operating as separate policy silos.

A lifetime model also respects patient reality. It acknowledges that non-response often reflects timing, barriers, fear, stigma, or lack of navigation rather than lack of concern. It responds by aligning invitations with readiness and risk, and by supporting completion rather than assuming it. It also makes equity operational: the model assumes barriers are normal and builds navigation and flexible delivery into routine care, rather than treating them as optional extras.

The most important next step is not to debate the value of the concept but to test it through implementation. A sensible programme of work would pilot life-course triggers in primary care, link them to registry-based task pathways, fund navigation, and evaluate outcomes that matter: completion, time-to-action, stage distribution in targeted groups, interval cancers, patient experience and equity impacts. Evaluation should also track unintended consequences-workload displacement, differential uptake by deprivation and ethnicity, and whether navigation improves completion without increasing inappropriate investigation. International experience suggests that integrated, registry-driven sequencing across life stages is feasible at scale (18, 19). The question for the UK is whether cancer control will continue to be designed primarily around episodic age triggers, or whether it will evolve into an accountable life-course system that is better matched to how risk accumulates and how people live.

If cancer control is a lifelong challenge, strategies designed as one-time events will continue to underperform. A lifetime approach does not reject age-based screening; it reinforces it by adding what the current model lacks: risk awareness, timing, navigation and completion. The promise is not perfection, but a measurable improvement in participation and earlier-stage detection among those currently left behind.

## References

[1] NHS England. Cancer statistics: incidence, 2022/23. Available online at: https://www.england.nhs.uk/statistics/statistical-work-areas/cancer-waiting-times/ (Accessed January 30, 2026).

[2] National Disease Registration Service (NDRS). Routes to Diagnosis. NHS England. Available online at: https://digital.nhs.uk/ndrs/data/data-outputs/cancer-data-hub/cancer-routes-to-diagnosis (Accessed January 30, 2026).

[3] Bradley SH. Increases in GP cancer referrals reflect successful health policy, not accidental overmedicalisation. BMJ. (2023) 381:1349. doi: 10.1136/bmj.p1349 37339813

[4] UK National Screening Committee. 2021 to 2022 data from England underlines huge impact of national NHS screening programmes. Screening Blog. Available online at: https://nationalscreening.blog.gov.uk/2025/01/30/2021-to-2022-data-from-england-underlines-huge-impact-of-national-nhs-screening-programmes/ (Accessed January 30, 2025).

[5] Cancer Research UK. Lung health check programme: progress and pilot results. 2024. Available online at: https://www.cancerresearchuk.org/about-cancer/lung-cancer/getting-diagnosed/screening (Accessed January 30, 2026).

[6] NHS England. Barriers to participation in cervical screening among 25–29-year-olds. South East Regional Public Health Screening. (2023). Available online at: https://www.england.nhs.uk/south-east/info-professionals/public-health/cervical-screening/6374-2/factors-influencing-25-29-year-olds-engagement-with-cervical-screening/barriers-to-participation/ (Accessed January 30, 2026).

[7] Cancer Research UK. Encouraging participation in bowel screening. London: Cancer Research UK for Health Professionals. (2023).

[8] OECD. Health at a Glance 2023: cancer screening. Paris: organisation for economic co-operation and development (2023). Available online at: https://www.oecd.org/en/publications/2023/11/health-at-a-glance-2023_e04f8239/full-report/cancer-screening_212c79aa.html (Accessed January 30, 2026).

[9] Eurostat. Cancer screening statistics – statistics explained. luxembourg: european commission (2024–2025). Available online at: https://ec.europa.eu/eurostat/statistics-explained/index.php/Cancer_screening_statistics (Accessed January 30, 2026).

[10] von Wagner C Baio G Raine R Snowball J Morris S Atkin W . Inequalities in participation in an organized national colorectal cancer screening programme: results from the first 26 million invitations in England. Int J Epidemiol. (2011) 40:712–8. doi: 10.1093/ije/dyr008 21330344

[11] Taratula-Lyons M Hill MC. Young women's perceptions of cervical screening in the UK: a qualitative study. Prim Health Care Res Dev. (2024) 25:e49. doi: 10.1017/S1463423624000446 39419821 PMC11569844

[12] International agency for research on cancer. World cancer report: cancer research for cancer prevention. Lyon: IARC (2020). Available online at: https://www.iarc.who.int/cards_page/world-cancer-report/ (Accessed January 30, 2026).

[13] Zhang L Carvalho AL Mosquera I Wen T Lucas E Sauvaget C . An international consensus on the essential and desirable criteria for an 'organized' cancer screening programme. BMC Med. (2022) 20:101. doi: 10.1186/s12916-022-02291-7 35317783 PMC8941752

[14] World Health Organization. Hepatitis B. Geneva: WHO (2023). Available online at: https://www.who.int/news-room/fact-sheets/detail/hepatitis-b (Accessed January 30, 2026).

[15] World Health Organization. Global strategy to accelerate the elimination of cervical cancer as a public health problem. Geneva: WHO (2020). Available online at: https://www.who.int/publications/i/item/9789240014107 (Accessed January 30, 2026).

[16] NHS England. NHS. Health Check: Best practice guidance. London: NHS England. (2023).

[17] Royal College of General Practitioners. Cancer diagnosis in primary care: a toolkit for GPs. London: RCGP (2022). Available online at: https://elearning.rcgp.org.uk/mod/book/tool/print/index.php?id=12890 (Accessed January 30, 2026).

[18] Australian Government Department of Health and Aged Care. National cervical screening program: self-collection policy (2023). Available online at: https://www.health.gov.au/initiatives-and-programs/national-cervical-screening-program (Accessed January 30, 2026).

[19] National Institute for Public Health and the Environment (RIVM). Cancer screening programmes in the Netherlands. Bilthoven: RIVM (2023). Available online at: https://www.rivm.nl/en/population-screening-programmes (Accessed January 30, 2026).

